# Hemodynamics of thrombus formation in intracranial aneurysms: An *in silico* observational study

**DOI:** 10.1063/5.0144848

**Published:** 2023-07-07

**Authors:** Qiongyao Liu, Ali Sarrami-Foroushani, Yongxing Wang, Michael MacRaild, Christopher Kelly, Fengming Lin, Yan Xia, Shuang Song, Nishant Ravikumar, Tufail Patankar, Zeike A. Taylor, Toni Lassila, Alejandro F. Frangi

**Affiliations:** 1 Centre for Computational Imaging and Simulation Technologies in Biomedicine (CISTIB), School of Computing, University of Leeds, Leeds, United Kingdom; 2 Leeds Institute for Cardiovascular and Metabolic Medicine (LICAMM), School of Medicine, University of Leeds, Leeds, United Kingdom; 3 EPSRC Centre for Doctoral Training in Fluid Dynamics, University of Leeds, Leeds, United Kingdom; 4 Leeds General Infirmary, Leeds, United Kingdom; 5 School of Mechanical Engineering, University of Leeds, Leeds, United Kingdom; 6 Department of Cardiovascular Sciences, KU Leuven, Leuven, Belgium; 7 Department of Electrical Engineering (ESAT), KU Leuven, Leuven, Belgium; 8 Alan Turing Institute, London, United Kingdom

## Abstract

How prevalent is spontaneous thrombosis in a population containing all sizes of intracranial aneurysms? How can we calibrate computational models of thrombosis based on published data? How does spontaneous thrombosis differ in normo- and hypertensive subjects? We address the first question through a thorough analysis of published datasets that provide spontaneous thrombosis rates across different aneurysm characteristics. This analysis provides data for a subgroup of the general population of aneurysms, namely, those of large and giant size (>10 mm). Based on these observed spontaneous thrombosis rates, our computational modeling platform enables the first *in silico* observational study of spontaneous thrombosis prevalence across a broader set of aneurysm phenotypes. We generate 109 virtual patients and use a novel approach to calibrate two trigger thresholds: residence time and shear rate, thus addressing the second question. We then address the third question by utilizing this calibrated model to provide new insight into the effects of hypertension on spontaneous thrombosis. We demonstrate how a mechanistic thrombosis model calibrated on an intracranial aneurysm cohort can help estimate spontaneous thrombosis prevalence in a broader aneurysm population. This study is enabled through a fully automatic multi-scale modeling pipeline. We use the clinical spontaneous thrombosis data as an indirect population-level validation of a complex computational modeling framework. Furthermore, our framework allows exploration of the influence of hypertension in spontaneous thrombosis. This lays the foundation for *in silico* clinical trials of cerebrovascular devices in high-risk populations, e.g., assessing the performance of flow diverters in aneurysms for hypertensive patients.

## I. INTRODUCTION

Spontaneous thrombosis (ST) of large and giant (>10 mm) unruptured intracranial aneurysms (IAs) is a common event that can be detected incidentally during advanced neuroradiological studies before treatment.^1–3^ These spontaneously thrombosed aneurysms are considered unstable dynamic structures that may grow, recanalize, bleed, compress, or cause thromboembolic events.^2–4^ Partially spontaneously thrombosed aneurysms may serve as a source of emboli leading to ischemic attack^3,5^ or cerebral infarction.^1,5^ Complete ST can sometimes stabilize the growth of the lesion, however, 33% (7/21) of the completely thrombosed aneurysms presented recanalization at follow-up.^1^

Given this, it is worth asking, what is the precise prevalence of ST formation in IAs? In the literature, spontaneous intra-aneurysmal thrombosis is a common phenomenon, and the ST prevalence reported in different studies varies widely. The ST prevalence rate of pediatric patients (8.3%–16.9%) is higher than that in adults,^6,7^ while female patients are more likely to present with ST than male patients.^1^ Similarly, differences are observed depending on the aneurysms themselves, with ST in small aneurysms (≤10 mm) being a much rarer phenomenon^1,8^ than the approximately 50% prevalence rate in giant IAs (≥25 mm).^3,9^ Despite the statistics, the prevalence of ST on a population level is not well-understood, as most studies had small sample sizes. We collect the clinically reported prevalence rates of ST (partial or complete) in IAs without treatment in the literature and conduct a statistical analysis to identify the prevalence rate of ST across different patient demographics and aneurysm characteristics.

Experimental studies have highlighted the importance of hemodynamic factors in the growth and rupture of aneurysms.^10,11^ However, *in vivo* or image-based population-level analysis of thrombosis hemodynamics in realistic anatomies and physiologies is currently very difficult, if not impossible. Computational modeling has proven to be a powerful tool in predicting thrombosis in aneurysms before and after treatment, and thus in patient-specific treatment planning or *in silico* trials,^12,13^ nonetheless, such an approach remains dependent on hemodynamic thresholds usually chosen based on ranges derived from limited highly controlled *in vitro* experiments, instead of accurate representatives of real anatomy and physiology. Computational fluid dynamics (CFD) models use hemodynamic surrogates in thrombosis initiation, with residence time (RT) and shear rate (SR) being the most widely used parameters in flow stasis-induced clotting models.^12,14,15^ However, there is no consensus on the trigger thresholds, with different values used throughout the literature.^12,14,16–18^ Therefore, there is a need to calibrate RT and SR thresholds for use in clotting models.^12^ In this study, we create, for the first time, an automatic computational workflow that enables population-level *in silico* studies to calibrate hemodynamic thresholds (RT and SR) of thrombus formation against real population-specific data.

Finally, we use our calibrated thrombosis model to study the effect of hypertension on ST. Hypertension is a well-known risk factor of unruptured IAs,^19^ and more than 75% of saccular IA patients have hypertension.^20^ The flow diverter performance assessment (FD-PASS) *in silico* trial showed that hypertension may cause less effective flow diversion,^13^ while the IntrePED study reported an association between hypertension and ischemic stroke in flow-diverted aneurysms.^21^ We modeled hypertension as boundary conditions modulated by a cerebral autoregulation system (CARS) model^22^ originally proposed by Mader *et al.*^23^

This paper aims to first establish the prevalence of ST in IAs with different characteristics and different demographics. We then calibrate the hemodynamic thresholds (RT and SR) of thrombus formation by matching our numerical ST prevalence to the real clinical data. Finally, we use our calibrated thrombosis model to investigate the differences in the ST prevalence and aneurysmal hemodynamic factors (RT and SR) in normotensive and hypertensive patients. The novelty of this study is that we create, for the first time, a fully automatic multi-scale modeling workflow that enables population-based *in silico* studies to calibrate hemodynamic thresholds (RT and SR) of thrombus formation against real population-specific data. We demonstrate how a mechanistic thrombosis model calibrated on an intracranial aneurysm cohort (large and giant IAs only) can help estimate ST prevalence in a broader aneurysm population (all sizes included). In addition, our framework can provide new insight into the impact of hypertension on thrombosis by modeling hypertension as boundary conditions modulated by a CARS model.^22^

## II. RESULTS

### A. Clinical ST prevalence

According to our search strategy, 434 studies were initially identified; after removing duplicates and case reports, 185 articles remained. Among these articles, 152 were excluded by title or abstract reading, while 33 underwent further detailed review for eligibility. A total of 11 studies were finally included in the statistical analysis.

In Table I, we present the summary of all ST prevalence calculated from the results of our literature review. The details of the collected cohort data of ST in large and giant aneurysms are shown in Tables S2 and S3. There are 646 IAs in total. The distribution of aneurysm sizes in these 646 IAs is as follows: 68% (437/646) small, 4% (29/646) large, and 28% (180/646) giant IAs. Of the 29 large IAs, seven cases presented with ST. Therefore, the ST prevalence (partial or complete) for large IAs is 24.1% (7/29)±7.9%, with 90% confidence. Of the 180 giant IAs, 97 cases were thrombosed. Therefore, the ST prevalence (partial and complete) for giant IAs is 53.9% (97/180)±6.1%, with 90% confidence.

**TABLE I.** Summary of the literature ST prevalence. ICA, internal carotid artery; MCA, middle cerebral artery; and BA, basilar artery.

Characteristics	ST prevalence	References
Giant (≥25 mm)	53.9%(97/180)±6.1%	3, 9, and 24–26
Large (>10 and <25 mm)	24.1%(7/29)±7.9%	24 and 26
Small (≤10 mm)	Rarely reported	1, 8, and 27
ICA (giant)	42.1%(32/76)±9.3%	3, 9, and 24–28
MCA (giant)	62.3%(33/53)±11.0%	3, 9, and 25
BA (giant)	63.2%(24/38)±12.9%	3, 9, and 25
Sex	Slight female prevalence	1
Age (<15 years)	8.3%–16.9%	6 and 7

For our simulation cohort with 40 large and 2 giant cases, according to Eq. (1), we estimated the ST prevalence rate as 25.5%. We used this as a criterion to calibrate the RT and SR threshold parameters.

### B. Numerical results of RT and SR

**FIG. 1.**
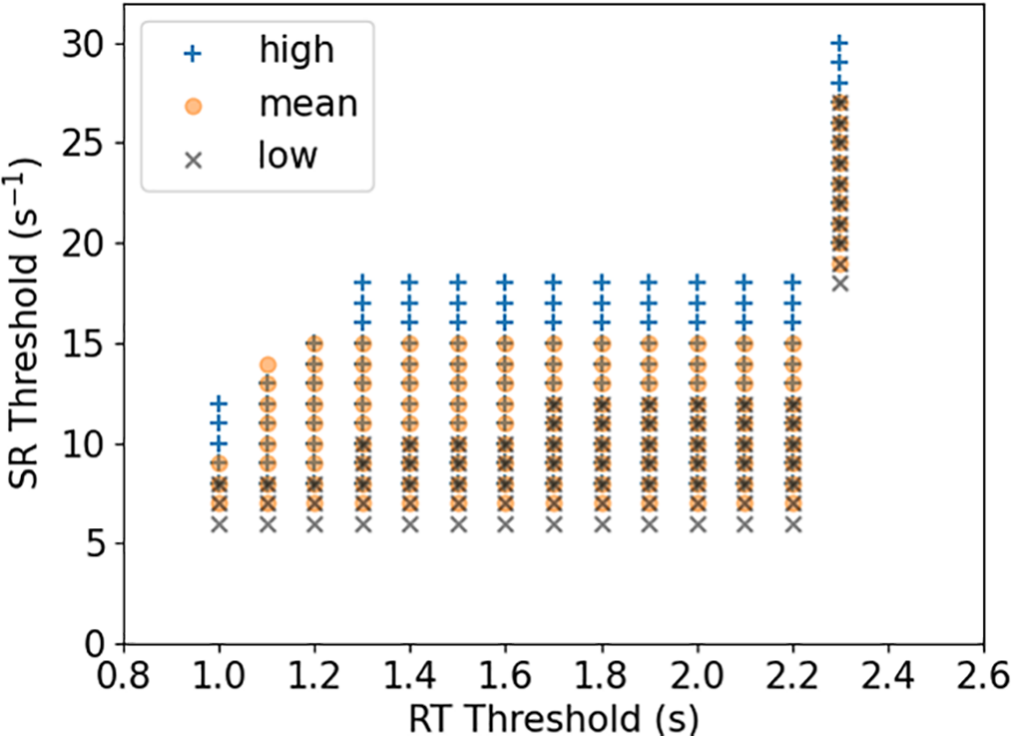
For a given case, if the RT is greater than the RT threshold and the SR is less than the SR threshold in the aneurysm sac, thrombus formation is assumed to have initiated. We plotted the scatter graph of the RT and SR thresholds that were found by matching our numerical ST prevalence to the clinical ST prevalence (25.5%) of large and giant aneurysms with a 5% tolerance. The step size of the RT threshold is 0.1 s, and the step size of the SR threshold is 1 s^−1^. The plausible threshold values for triggering thrombosis formation are those located in the overlap area.

The maximum RT and minimum SR in the aneurysm sac at mean flow normotensive conditions for all 42 large and giant cases are shown in Table S4. For a given pair of RT and SR thresholds, we obtained a specific numerical ST prevalence for large and giant IAs. We plotted all combinations of RT and SR threshold values that can make the simulated ST prevalence close (within ±5%) to the clinical ST prevalence of 25.5% (Fig. 1). The overlap of these parameters at high, mean, and low is considered to be the plausible range of thresholds since it indicates where the thresholds are largely independent of the inter-subject flow variability. We have 42 large and giant cases in total, and an increase or decrease in one thrombosed case increases or decreases the ST prevalence by 2.4%. In Fig. 1, we set a 5% tolerance for the numerical ST prevalence (tolerance for two cases), so the threshold values in the overlap region in Fig. 1 make the numerical ST prevalence between 20.5% and 30.5%. For the overlap area in Fig. 1, the RT and SR thresholds are in the ranges [1.0, 2.3] s and [8,27] s^−1^, respectively. The average values of RT and SR thresholds in the overlap area are 1.9 s and 11 s^−1^, respectively.

When using the average values of RT and SR thresholds, i.e., 1.9 s and 11 s^−1^, the numerical ST prevalence of large and giant IAs for our cohort is 28.6% (12/42)±11.5%, while the numerical ST prevalence of small IAs for our cohort is 17.9% (12/67)±7.7%. We estimated the ST prevalence of large IAs ( 24.1%±7.9%) and giant IAs ( 53.9%±6.1%) from the literature data and obtained the ST prevalence of small IAs ( 17.9%±7.7%) from our observational study. The distribution of aneurysm sizes in a general population with 1993 IAs^30^ is 68% (1355/1993) small IAs, 30% (598/1993) large IAs, and 2% (40/1993) giant IAs. Therefore, the ST prevalence of the general population containing all sizes of intracranial aneurysms can be estimated by Eq. (1) as 20.5%(408/1993)±1.5%.

Our simulation results show that bigger aneurysms are more likely to be thrombosed, which is consistent with the literature data.^1,8,27,31^ No exact ST prevalence for small IAs is reported in the literature. From a hemodynamics point of view, our study found that the numerical ST prevalence for small IAs of our cohort is 17.9%. Those thrombosed small IAs have a significantly higher aspect ratio (AR), measured as aneurysm height-to-neck width, than the non-thrombosed small IAs (Table II).

**TABLE II.** Comparison between thrombosed and non-thrombosed aneurysms. ICA, internal carotid artery; PCoA, posterior communicating artery; MCA, middle cerebral artery; BA, basilar artery; PCA, posterior cerebral artery; SCA, superior cerebellar artery; and ACA, anterior cerebral artery.

	ST large/giant	Non-ST large/giant	p value	ST small	Non-ST small	p value
Number of IAs	12	30		12	55	
Age (year)	51.9 ± 5.7	51.5 ± 5.8	0.83	50.5 ± 3.3	51.4 ± 7.5	0.55
Female, % (n/N)	58.3 (7/12)	66.7 (20/30)	0.35	66.7 (8/12)	72.7 (40/55)	0.84
Size (mm)	15.6 ± 6.1	13.1 ± 3.5	0.19	6.9 ± 1.0	6.1 ± 1.8	0.09
Neck width (mm)	6.3 ± 2.6	6.9 ± 2.1	0.49	3.4 ± 0.6	4.0 ± 1.1	0.01
Aspect ratio	1.9 ± 0.4	1.6 ± 0.7	0.05	1.7 ± 0.4	1.1 ± 0.5	8.2 × 10^−4^
Non-sphericity	0.26 ± 0.05	0.19 ± 0.06	4.5 × 10^−4^	0.26 ± 0.03	0.16 ± 0.06	1.7 × 10^−4^
Max RT (s)	2.51 ± 0.14	1.83 ± 0.72	2.1 × 10^−5^	2.46 ± 0.20	0.73 ± 0.60	4.1 × 10^−24^
Min SR (s^−1^)	4.80 ± 3.10	26.52 ± 16.36	5.2 × 10^−8^	3.23 ± 2.76	81.30 ± 102.52	8.0 × 10^−7^
Aneurysm location						
ICA/PCoA, % (n/N)	25.0 (3/12)	76.7 (23/30)		66.7 (8/12)	61.8 (34/55)	
MCA/Sylvian, % (n/N)	16.7 (2/12)	16.7 (5/30)		16.7 (2/12)	25.4 (14/55)	
BA/PCA/SCA, % (n/N)	50.0 (6/12)	6.6 (2/30)		8.3 (1/12)	7.3 (4/55)	
ACA, % (n/N)	8.3 (1/12)	0 (0/30)		8.3 (1/12)	5.5 (3/55)	
p values were computed using the two-tailed t-test.						

To investigate why bigger aneurysms are more likely to present with ST, we compared the demographics and aneurysm characteristics for thrombosed large and giant IAs, non-thrombosed large and giant IAs, thrombosed small IAs, and non-thrombosed small IAs (Table II). The above grouping for all 109 cases is based on the model-predicted ST status. These four groups are very similar in terms of patient age and sex, but the size and AR of the thrombosed groups are larger than that of the non-thrombosed groups; 62.5% (15/24) of the thrombosed group are high AR (>1.6)^13^ IAs. In contrast, high AR cases only account for 24.7% (21/85) of the non-thrombosed group. In our cohort, bigger aneurysms are also more likely to be high AR cases, with 59.5% (25/42) of the large and giant IAs having a high AR, while only 16.4% (11/67) of the small IAs have a high AR. The thrombosed groups, including both large/giant and small IAs, have a significantly higher AR and non-sphericity than the non-thrombosed groups. Our results show that IAs with higher AR and non-sphericity are more likely to be thrombosed and bigger aneurysms usually have a higher AR.

We also analyzed the relationships between aneurysm geometry characteristics and the two hemodynamic factors, RT and SR, for all 109 cases. As shown in Fig. S3, correlation between size and RT/SR is weaker than between AR and RT/SR, and the maximum RT increases with AR. The minimum SR in the aneurysm sac decreases with both size and AR.

### C. The effect of hypertension on the ST prevalence

**FIG. 2.**
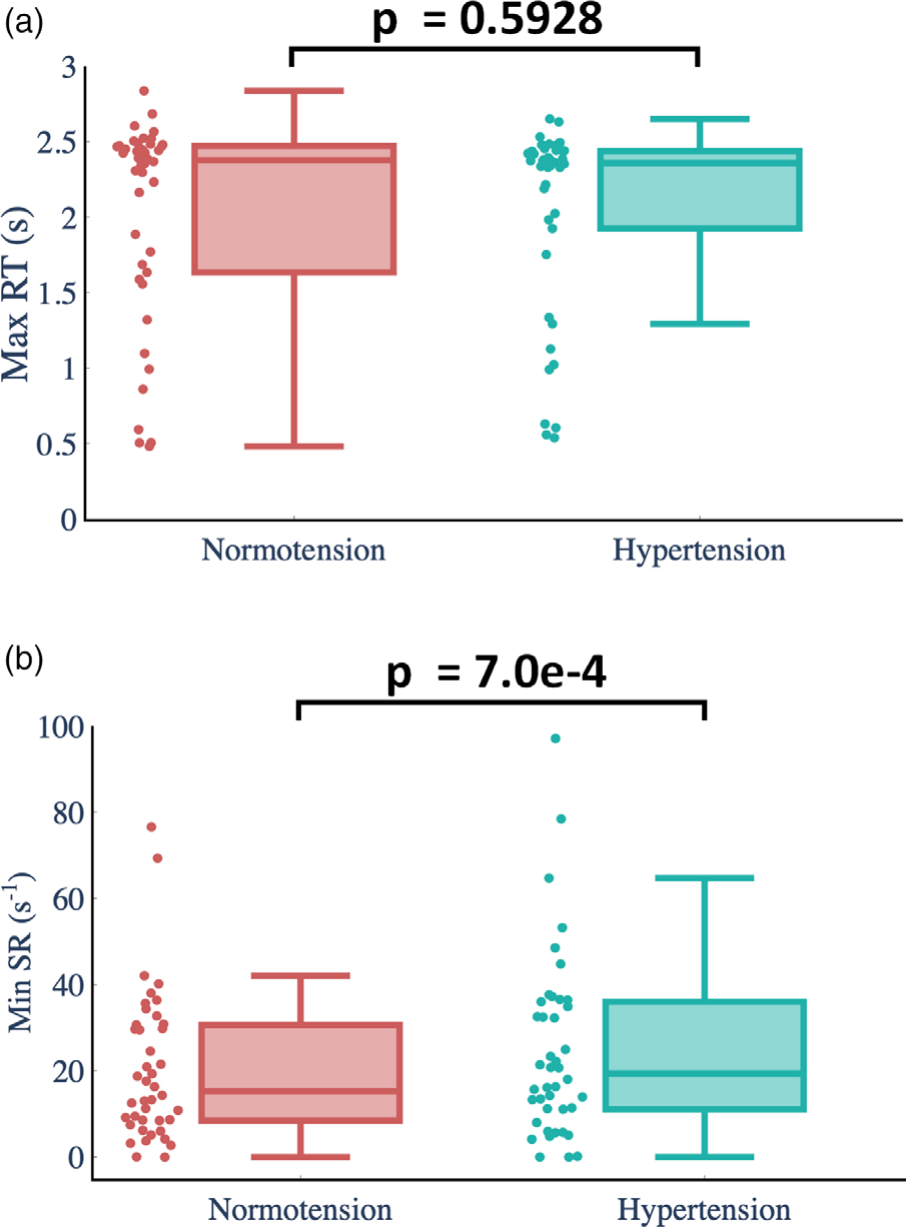
Comparison between normotension and hypertension for all 42 large and giant IAs in terms of the maximum RT and the minimum SR. In the box and whiskers plot, the horizontal line within the box denotes the median value. The lower and upper edges of the box denote the 25th and 75th percentiles. The ends of the whiskers are drawn to the upper and lower extreme values. p values were computed using the two-tailed t-test. (a) Maximum Residence Time (RT) and (b) Minimum Shear Rate (SR).

We imposed hypertensive inlet waveforms to investigate how hypertension affects the ST prevalence in large and giant IAs. Our numerical results show that the ST prevalence of large and giant IAs of our hypertension cohort is 23.8%, which is lower than at normotension (28.6%). To investigate why ST might be less common in hypertensive patients, we compared normotension and hypertension in terms of the maximum RT and minimum SR in the aneurysm sac for all 42 large and giant IAs (Fig. 2). No significant differences are observed in the maximum RT calculated under normotensive and hypertensive conditions (p = 0.5928). However, 90.5% (38/42) of cases in the hypertension cohort have a higher minimum SR than those in normotension cohort (p = 0.0007). This explains why flow stasis-induced computational models show a relatively low ST prevalence in hypertensive patients.

## III. DISCUSSION

The prevalence of ST in large and giant aneurysms reported in the literature varies widely. ST occurs in 10%–30% of unruptured large and giant aneurysms,^1^ while the prevalence of ST varies from 30% to 70%^1,3,9,27^ in giant aneurysms alone. For a specific cohort, the distribution of large and giant IAs can vary. The ST prevalence may be higher with a larger proportion of giant cases. This might be one of the main reasons why the ST prevalence in large and giant IAs varies widely. Another reason is that some statistics are based on limited sample sizes. To obtain more accurate ST prevalence in aneurysms of different sizes, we conducted a comprehensive literature review. We collected 646 cases in the literature, with 437 small, 29 large, and 180 giant IAs. 7 out of 29 large IAs and 97 out of 180 giant IAs spontaneously thrombosed; therefore, the ST prevalence is 24.1%(7/29)±7.9% for large IAs and 53.9%(97/180)±6.1% for giant IAs, with 90% confidence. These were used as benchmarks to calculate the ST prevalence of large and giant IAs according to the distribution of large and giant IAs in our simulation cohort. Although large and giant IAs are more likely than small IAs to be spontaneously thrombosed, the intraluminal thrombosis is present in approximately the same proportion of giant aneurysms, regardless of location.^25^ The ST prevalence of large and giant IAs in different locations (ICA, MCA, and BA) is shown in Table I, but these statistics are not included in our calibration experiment as it is well-established that aneurysm size^1,8,27^ is the main factor associated with ST, irrespective of its location.^25^

Three main triggers of flow stasis-based clotting models appear in the literature: (1) high RT alone; (2) low SR or wall shear stress (WSS) alone; and (3) high RT and low SR/WSS in combination. However, there is no clear consensus on the values of the trigger thresholds, and published works studying ST hemodynamic thresholds often have small sample sizes. Based on CFD simulations conducted on 113 aneurysms—where particles injected into the parent vessel were tracked over multiple cardiac cycles—Leemans *et al.*^32^ found that the particle RT for four out of five partially thrombosed aneurysms had a long RT (>1.9 s). While thrombus formation has been reported to initiate as early as RT = 1 s,^18^ Marsh *et al.*^33^ found in their experiments that a threshold of RT ≥ 1 s was ineffective as multiple patients in their cohort demonstrated a median RT >1 s in the aneurysm, even before treatment. Reza and Arzani^34^ critically compared different RT measures in aneurysms. They compared particle RT, Eulerian RT, and virtual-ink RT (*RT_V I_*) in one cerebral aneurysm geometry and obtained maximum RT values in the aneurysm sac of 0.66, 1.05, and 6.25 s, respectively. Rayz *et al.*^14^ estimated *RT_V I_* and analyzed the distribution of RT for three cases, for which they found that the mean RT was 18.22 ± 11 s (range 0.63–40.13 s) in the thrombosed area. Their results showed that a model with both low WSS and high RT could predict thrombosed areas significantly better than the models using RT or WSS alone. WSS correlates with SR through the viscosity of the fluid; however, SR is a measure of total blood deformation, which is a better indication of blood stagnation and potential thrombosis because it takes into account the shear forces due to both the wall and the surrounding fluid.^16^ Based on experimental results from Nyilas *et al.*,^35^ Gorring *et al.*^16^ assumed that flow-induced thrombosis would be initiated when SR <10 s^−1^; however, a limitation of their work is that simulations were based on an idealized cavity rather than a real aneurysm geometry. Based on a Fourier analysis of SR at thrombosed locations in the aneurysm sac of 10 CFD simulations, de Sousa *et al.*^17^ determined 25 s^−1^ as the threshold for the initiation of thrombosis.

High RT and low SR are usually used in computational models to characterize the flow stasis that triggers the thrombosis formation process in the aneurysm sac. We calibrated RT and SR thresholds using the clinical ST prevalence of large and giant IAs. Following calibration, the plausible range of RT and SR thresholds for our model is located in the overlap area in Fig. 1, which indicates that the corresponding simulation results are largely independent of the inter-subject flow variability. As shown in Table III, the RT threshold is in the range [1.0, 2.3] s, with an average value of 1.9 s; the SR threshold is in the range [8,27] s^−1^, with an average value of 11 s^−1^. The calibrated RT and SR thresholds are consistent with the literature and also more reliable due to the rigorous calibration based on clinical data.

**TABLE III.** Comparison between our CFD calibrated thresholds and the literature thresholds.

Results	Simulation	Literature
RT threshold (s)	1.9 (range: 1–2.3)	1–5 (Refs. 12, 14, 18, 32, and 33)
SR threshold (s^−1^)	11 (range: 8–27)	10–25 (Refs. 12, 16, and 17)

ST is not a well-documented phenomenon in hypertensive patients. This might be because most patients treated for aneurysms have blood pressure-controlling measures in place.^36^ Using the calibrated RT and SR threshold values, our model predicted a slightly lower ST prevalence for our virtual cohort in hypertensive conditions, modeled as boundary conditions from a CARS model.^22^ As shown in Fig. 2, the minimum SR in the aneurysm sac in hypertension is usually higher than in normotension (P <0.001), although the maximum RT is relatively similar (p = 0.5928). SR is sensitive to changes in normotensive and hypertensive conditions, whereas RT is robust. Blood flow stasis-induced models are usually triggered by high RT and low SR. In a specific area in the aneurysm sac, only if RT is higher than the RT threshold and SR is lower than the SR threshold is the clotting process assumed to have initiated. From our simulation results, the hypertensive conditions have limited effect on RT and SR in the aneurysm sac, thus only causing a slightly lower ST prevalence than is found in normotensive patients.

We performed CFD simulations for 109 cases and analyzed the relationship between aneurysm geometry characteristics and flow, finding that bigger aneurysms usually have a higher AR in our cohort (Table II). Higher AR and more complex shape (higher non-sphericity) lead to higher RT and lower SR in the aneurysm sac, and high RT and low SR ultimately trigger the thrombosis formation. There is a strong link between IA morphology and hemodynamics.^17^ The high prevalence of thrombus formation within large and giant IAs is related to AR.^8^ Although high AR is not sufficient for reliable prediction of thrombus formation, IAs with higher AR are more likely to present with ST. High AR results in low velocity, low SR, and inhibition of pulsatile blood flow (Figs. S3–S6), leading to a pro-inflammatory and pro-coagulable micro-environment at the aneurysm wall.^1,17^

### A. An example case comparison between normotension and hypertension

In this section, we show how hypertension affects the distribution and magnitude of RT and SR with an example case. We performed six simulations for each virtual case using six different inlet flow waveforms. A middle-aged female patient case (51 years old; aneurysm size, 17.8 mm; AR, 2.3; location, basilar tip) was selected for a detailed analysis. The inlet flow shown in Fig. S2, which is generated from a MGM model^29^ and a CARS model^22^ according to the age and gender of the patient, is imposed for simulation for this case. As shown in Fig. S7, the time-averaged velocity distributions are roughly similar for normotension and hypertension, although the velocity magnitude is slightly higher for normotension. This is because although the peak velocity of the hypertensive inlet flow waveform is higher than that of the normotensive inlet flow waveform, the mean velocity is higher for normotension (mean: 13.78 cm/s) than for hypertension (mean: 11.29 cm/s).

**FIG. 3.**
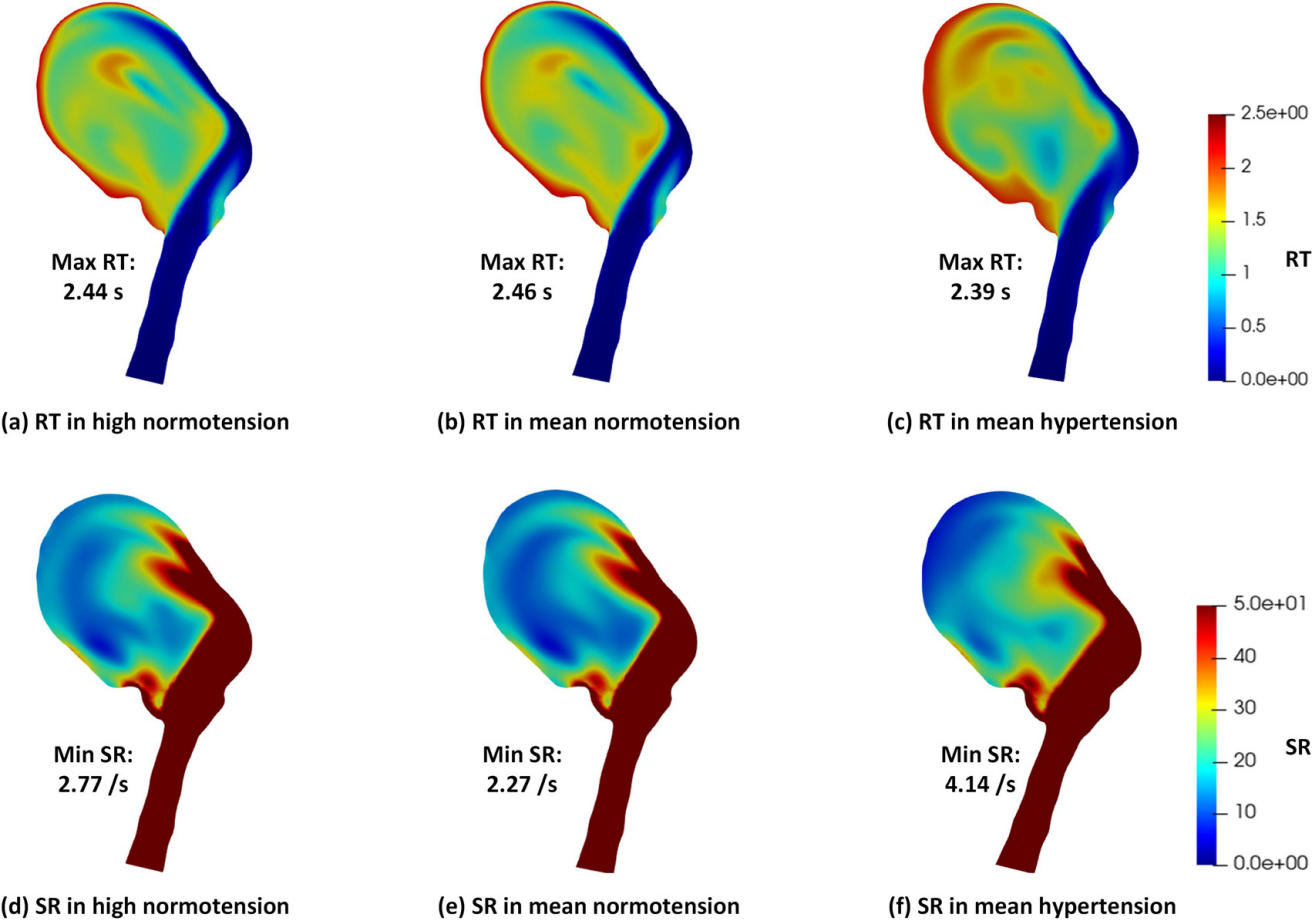
Residence time (RT) and shear rate (SR) distribution in normotensive and hypertensive conditions.

For a given state (normotension/hypertension), the distribution and magnitude of RT and SR are almost equivalent. For example, in Fig. 3, the results of Figs. 3(a) and 3(b) are very similar. The difference between the maximum RT values in normotensive and hypertensive conditions is small, whereas the distribution of RT differs. We found apparent differences in both the magnitude and distribution of SR from normotension to hypertension. In this case, slightly lower maximum RT and higher minimum SR in the aneurysm sac are observed in hypertensive conditions.

### B. Limitations

The following are the limitations of this study: (1) we assumed clotting in the aneurysm sac was triggered by blood flow stasis, and high RT and low SR were usually used in computational models to characterize the flow stasis. Therefore, we only focused on studying RT and SR in this paper. (2) Although we performed a systematic review, we did not find enough data to estimate ST prevalence for small aneurysms. It is well-established that aneurysm size is the most important factor associated with ST, and this phenomenon in large and giant aneurysms is well-documented. We only used the clinical ST prevalence of large and giant IAs to calibrate our clotting model. (3) There were few ST cases reported in hypertensive patients for us to use to estimate the ST prevalence and compare our results. This might be because most patients treated for aneurysms have blood pressure-controlling measures in place. Ours is the first study that looked into RT and SR for hypertensive patients. Our results showing a slightly lower prevalence of ST in hypertensive cases were based only on the hemodynamics point of view, and there may be other physiology involved, which was not included in our analysis.

### C. Conclusion

In this sensitivity analysis into stasis-driven thrombosis trigger thresholds, we first conducted a systematic literature review to estimate the clinical ST prevalence rate for a subgroup of the general population of aneurysms, namely, those of large and giant size (>10 mm). We then performed a series of numerical simulations for a virtual cohort of 109 patients and used the estimated clinical ST prevalence of large and giant IAs as a criterion to calibrate the trigger thresholds in thrombosis models. We showed how clotting models can be calibrated on aneurysm cohort and then help to estimate the ST prevalence for a general population. To accelerate this *in silico* calibration experiment, we created a fully automatic workflow to segment the image data, generate the volume mesh, impose patient-specific boundary conditions, and run the simulations on a cloud computing platform. Our results showed that the high prevalence of thrombus formation within large and giant IAs is related to high AR. Bigger aneurysms usually have a higher AR, and IAs with higher AR are more likely to be thrombosed. Our calibration experiment identified the plausible values of two commonly used thrombosis trigger threshold parameters, RT and SR, as 1.9 s and 11 s^−1^, respectively. Furthermore, our model predicted a slightly lower ST prevalence in hypertensive than in normotensive patients due to the larger minimum SR in the aneurysm sac caused by hypertension. We found that SR is sensitive to the changes of boundary conditions from normotension to hypertension, while RT is more robust. This study not only collated ST literature and demonstrated how clinical ST prevalence data could guide computational thrombus formation modeling by identifying plausible ranges of model parameters but also revealed that ST may be less common in hypertensive patients with large and giant IAs.

## IV. METHODS

**FIG. 4.**
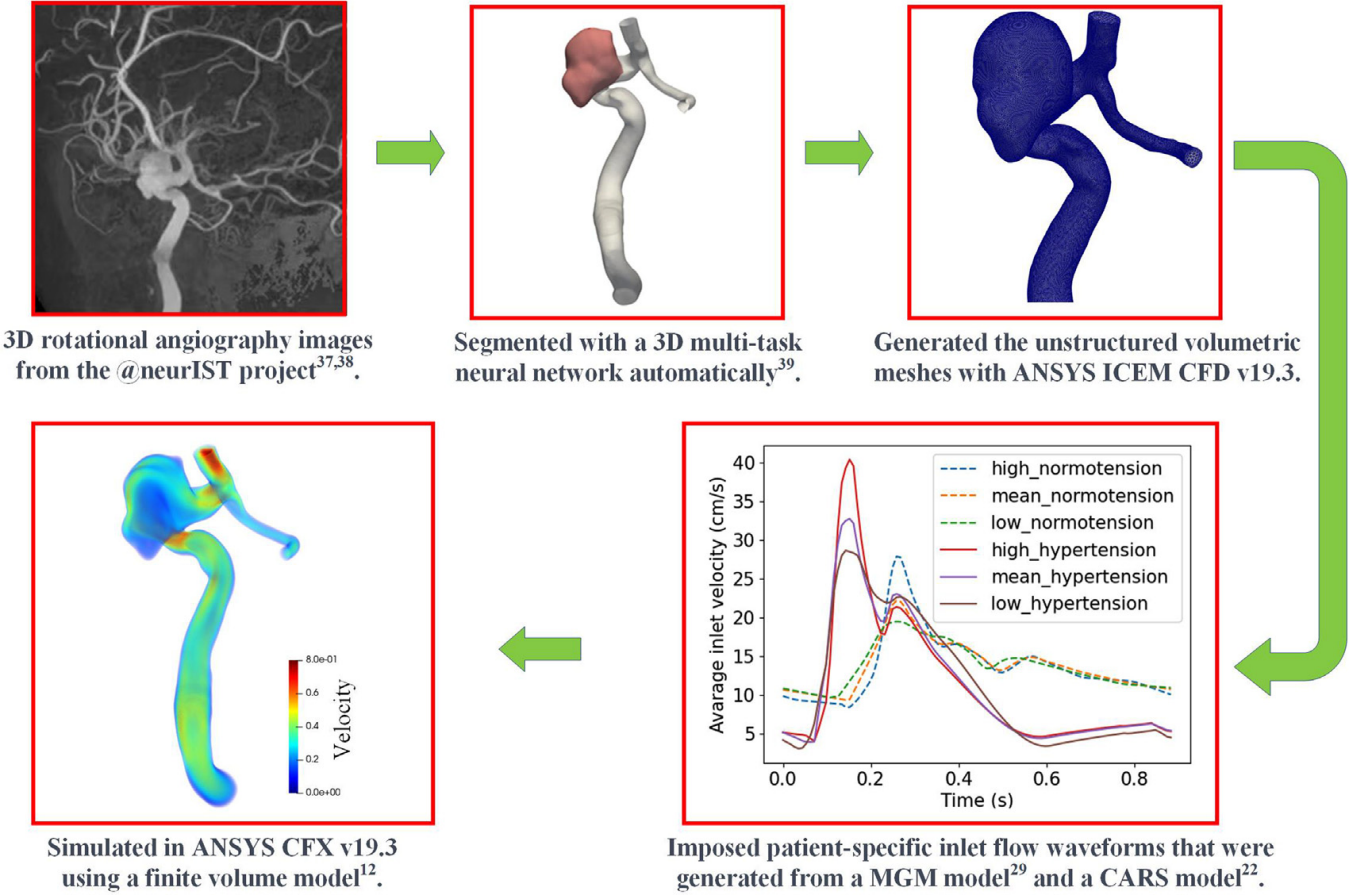
Automatic workflow on MULTI-X. The images are from the @neurIST project.^37,38^ We applied a multi-tasking neural network to automatically segment these images^39^ and generate the corresponding patient-specific vascular surface models, and then we used ANSYS ICEM CFD v19.3 (Ansys Inc. Canonsburg, PA, USA) to generate unstructured volumetric meshes. The patient-specific inlet flow waveforms were generated from a multivariate Gaussian model (MGM)^29^ and a CARS model.^22^ Finally, the coupled Navier–Stokes equations and transport equation for RT were solved in ANSYS CFX v19.3 (Ansys Inc., Canonsburg, PA, USA) using a finite volume method.^12^

Through a thorough analysis of published datasets that provided ST rates for a subgroup of the general population of aneurysms, namely, those of large and giant aneurysms (>10 mm), we estimated the clinical ST prevalence of large and giant IAs for our simulation cohort. Using the clinical ST prevalence rate, we performed an *in silico* observational study in 109 virtual patients to calibrate RT and SR thresholds and estimate ST prevalence in a broader IA population. We further investigated how ST differs in normo- and hypertensive conditions. As shown in Fig. 4, we created a fully automatic workflow executed on a cloud computing platform, MULTI-X (https://multi-x.org), that segments angiographic image data, generates volumetric meshes, sets patient-specific boundary conditions using a statistical population model, assembles and executes fluid dynamic simulations, and provides patient-specific intra-aneurysmal flows. While each component of our methodological framework has been independently developed and validated before, this study is the first to model and present such a complex process on the largest patient cohort to date.

### A. Systematic literature review

To estimate the ST prevalence for different aneurysm characteristics, a comprehensive and systematic review of the literature up to July 2021 was conducted on the MEDLINE database. We searched for articles reporting cohort cases of IA ST (partial or complete). The articles were identified using Boolean searches on PubMed with the following keywords “[(spontaneous thrombosis) or (spontaneous clot formation)] and [(intracranial aneurysms) or (cerebral aneurysms)].” The search strategy followed the PRISMA (Preferred Reporting Items for Systematic Review and Meta-Analyses) guidelines.^40^ Case report articles were not considered. Only articles in English were considered, and only those reporting patient cohorts. Aneurysms were divided into three groups: small (≤10 mm), large (>10 and <25 mm), and giant (≥25 mm) according to the sizes reported by the investigators.

After the systematic literature review, we were able to estimate the ST prevalence of aneurysms of different sizes. For a population, the ST prevalence (*P*) is given by

$$P=\frac{N_{s}P_{s}+N_{l}P_{l}+N_{g}P_{g}}{N_{s}+N_{l}+N_{g}}\times 100\%,$$ (1)

where *N_s_*, *N_l_*, and *N_g_* are the numbers of small, large, and giant IAs, respectively. *P_s_*, *P_l_*, and *P_g_* are the ST prevalence of small, large, and giant IAs, respectively. However, ST is rarely reported in small IAs, so we used the collected ST prevalence of only large and giant IAs, *P_l_* and *P_g_*, to estimate the clinical ST prevalence for our cohort according to its distribution of large and giant cases. We calibrated our clotting model by matching the numerical ST prevalence to the estimated clinical ST prevalence for our simulation cohort. After calibration, we used the calibrated model to predict the ST prevalence for small aneurysms, *P_s_*. Finally, the ST prevalence for a broader general population can be estimated by Eq. (1).

### B. *In silico* observational study

#### 1. Patient and aneurysm characteristics

The patient image data in this paper were from the @neurIST project.^37,38^ All images were anonymized, respecting the @neurIST ethical approval for use of patient data (http://www.aneurist.org). We only considered single aneurysm cases. A total of 109 patient datasets (72 females, 37 males), with 67 small, 40 large, and 2 giant IAs, were available. Among the 109 cases, 67 small, 21 large, and 1 giant cases were segmented automatically, while the other 19 large and 1 giant cases were manually segmented in the @neurIST project. The details of the population characteristics of our simulation cohort can be found in the supplementary material Table S1; the average age of our simulation cohort is 51 years (range 22–78 years), and the mean aneurysm neck size is 5.0 ± 2.1 mm (range 1.7–12.3 mm). Of these 109 IAs, 61% (67/109) are small IAs, 37% (40/109) are large IAs, and 2% (2/109) are giant IAs. It is well-established in the literature that aneurysm size is the most important factor associated with ST.^1,8,27^ The distribution of aneurysm sizes in our simulation cohort is quite similar to that of the general population obtained from a large consecutive series of 1993 ruptured IAs:^30^ 68% (1355/1993) small IAs, 30% (598/1993) large IAs, and 2% (40/1993) giant IAs. Thus, the size-based ST prevalence calculated by our calibrated model can be applied to the general population.

#### 2. Flow simulations

Multiple steps are required to generate volumetric meshes for CFD simulations. We used a 3D multi-task segmentation neural network^39^ to complete the segmentation of the vessel and aneurysm dome automatically. This segmentation network achieved an average Dice score of 0.82 and average surface-to-surface error of 0.20 mm [less than the in-plane resolution (0.35 mm/pixel)]; from the segmentation, we acquired patient-specific vascular surface models that were used to generate unstructured volumetric meshes using ANSYS ICEM CFD v19.3 (Ansys Inc. Canonsburg, PA, USA). Details of the mesh convergence analysis, simulation specifications, and equations for calculating RT and SR can be found in the supplementary material.

For a given case, if RT is greater than the RT threshold and SR is less than the SR threshold in the aneurysm sac, thrombus formation is assumed to initiate. We performed a series of unsteady simulations using the generated volumetric meshes and calculated the magnitude and distribution of RT and SR on the whole computational domain (both the vessel and the aneurysm sac) and on the isolated aneurysm sac for each case (Fig. S1). By varying the values of the RT and SR thresholds, we obtained different numerical ST prevalence across our cohort of large and giant IAs. Finally, by matching the simulated ST prevalence in our large and giant IA cohort to the clinical ST prevalence, we were able to obtain a plausible range of trigger threshold values for RT and SR and calibrate our model.

#### 3. Patient-specific inlet flow boundary conditions

Previous studies have found that inlet flow boundary conditions of CFD models affect IA hemodynamics, with inter-subject variability in cerebral blood flow found to be 10%–20%.^22,41–43^ With *in vivo* measurement-derived boundary conditions unavailable for our cohort, we used a previously developed multivariate Gaussian model (MGM)^29^ to generate patient-specific boundary conditions as internal carotid flow waveforms. The MGM model was trained and calibrated using data from 17 healthy young adults.^44^ A virtual population of 1000 normotensive waveforms was then generated, while three of them, i.e., high, mean, and low, were selected to maximize the variability across the entire virtual population (by selecting high/low as the upper/lower bounds of the 1000 waveforms). To consider inter-subject variability, these three representative waveforms were used as inlet boundary conditions for flow simulations. Poiseuille's law was used to scale the MGM-generated waveforms to achieve a time-averaged wall shear stress of 1.5 Pa at the inlet. Figure S2 shows the inlet flow waveforms for a 51-year-old female.

According to the latest definition of hypertension from the American College of Cardiology/American Heart Association (ACC/AHA) in November 2017,^45^ participants are considered hypertensive if they have a measured systolic blood pressure SBP ≥ 130 mm Hg or a measured diastolic blood pressure DBP ≥ 80 mm Hg. We use a computational model of cerebral autoregulation^22^ originally proposed by Mader *et al.*^23^ to model the effect of hypertension on IA hemodynamics. The CARS model takes the normotensive blood flow waveforms calculated for each virtual case, estimates the corresponding normotensive pressure waveforms (SBP: 90–120 mm Hg, DBP: 70–80 mm Hg, heart rate: 68 bpm), scales the normotensive pressure waveforms to hypertensive waveforms (fixed scale factor 1.3 of SBP and DBP,^22,46^ 68 bpm), and finally generates the associated hypertensive waveforms.

## SUPPLEMENTARY MATERIAL

See the supplementary material for the governing equations, simulation specifications, mesh convergence analysis, details of the literature data, and other additional materials.

## Data Availability

The data that support the findings of this study are available from the corresponding author upon reasonable request.

## References

[1] V. Alberto , M. Mahmoud , S. Daniele , P. Francesco , D. R. Thomas , and C. Giulia , “ Complete spontaneous thrombosis in unruptured non-giant intracranial aneurysms: A case report and systematic review,” Clinical Neurol. Neurosurg. 200, 106319 (2020). 10.1016/j.clineuro.2020.106319 33268195

[2] J. E. Cohen , E. Yitshayek , J. M. Gomori , S. Grigoriadis , G. Raphaeli , S. Spektor , and G. Rajz , “ Spontaneous thrombosis of cerebral aneurysms presenting with ischemic stroke,” J. Neurol. Sci. 254, 95–98 (2007). 10.1016/j.jns.2006.12.008 17258773

[3] I. Whittle , N. W. Dorsch , and M. Besser , “ Spontaneous thrombosis in giant intracranial aneurysms,” J. Neurol., Neurosurg. Psychiatry 45, 1040–1047 (1982). 10.1136/jnnp.45.11.1040 7175528 PMC491643

[4] J. E. Cohen , G. Rajz , F. Umansky , and S. Spektor , “ Thrombosis and recanalization of symptomatic nongiant saccular aneurysm,” Neurol. Res. 25, 857–859 (2003). 10.1179/016164103771953961 14669530

[5] R. D. Brownlee , B. I. Tranmer , R. J. Sevick , G. Karmy , and B. J. Curry , “ Spontaneous thrombosis of an unruptured anterior communicating artery aneurysm: An unusual cause of ischemic stroke,” Stroke 26, 1945–1949 (1995). 10.1161/01.STR.26.10.1945 7570753

[6] P. Lasjaunias , S. Wuppalapati , H. Alvarez , G. Rodesch , and A. Ozanne , “ Intracranial aneurysms in children aged under 15 years: Review of 59 consecutive children with 75 aneurysms,” Child's Nervous Syst. 21, 437–450 (2005). 10.1007/s00381-004-1125-x 15834727

[7] J. Liang , Y. Bao , H. Zhang , K. H. Wrede , X. Zhi , M. Li , and F. Ling , “ The clinical features and treatment of pediatric intracranial aneurysm,” Child's Nervous Syst. 25, 317–324 (2009). 10.1007/s00381-008-0725-2 18839183

[8] H. Ohta , N. Sakai , I. Nagata , H. Sakai , A. Shindo , and H. Kikuchi , “ Spontaneous total thrombosis of distal superior cerebellar artery aneurysm,” Acta Neurochir. 143, 837–843 (2001). 10.1007/s007010170039 11678406

[9] O. Schubiger , A. Valavanis , and J. Hayek , “ Computed tomography in cerebral aneurysms with special emphasis on giant intracranial aneurysms,” J. Comput. Assisted Tomogra. 4, 24–32 (1980). 10.1097/00004728-198002000-00005 7354171

[10] H. Artmann , D. Vonofakos , H. Müller , and H. Grau , “ Neuroradiologic and neuropathologic findings with growing giant intracranial aneurysm. Review of the literature,” Surg. Neurol. 21, 391–401 (1984). 10.1016/0090-3019(84)90120-4 6367118

[11] H. Meng , V. Tutino , J. Xiang , and A. Siddiqui , “ High WSS or low WSS? complex interactions of hemodynamics with intracranial aneurysm initiation, growth, and rupture: Toward a unifying hypothesis,” Am. J. Neuroradiol. 35, 1254–1262 (2014). 10.3174/ajnr.A3558 23598838 PMC7966576

[12] A. Sarrami-Foroushani , T. Lassila , S. M. Hejazi , S. Nagaraja , A. Bacon , and A. F. Frangi , “ A computational model for prediction of clot platelet content in flow-diverted intracranial aneurysms,” J. Biomech. 91, 7–13 (2019). 10.1016/j.jbiomech.2019.04.045 31104921

[13] A. Sarrami-Foroushani , T. Lassila , M. MacRaild , J. Asquith , K. C. Roes , J. V. Byrne , and A. F. Frangi , “ In-silico trial of intracranial flow diverters replicates and expands insights from conventional clinical trials,” Nat. Commun. 12, 3861 (2021). 10.1038/s41467-021-23998-w 34162852 PMC8222326

[14] V. Rayz , L. Boussel , L. Ge , J. Leach , A. Martin , M. Lawton , C. McCulloch , and D. Saloner , “ Flow residence time and regions of intraluminal thrombus deposition in intracranial aneurysms,” Ann. Biomed. Eng. 38, 3058–3069 (2010). 10.1007/s10439-010-0065-8 20499185 PMC2940011

[15] R. Ouared , I. Larrabide , O. Brina , P. Bouillot , G. Erceg , H. Yilmaz , K.-O. Lovblad , and V. M. Pereira , “ Computational fluid dynamics analysis of flow reduction induced by flow-diverting stents in intracranial aneurysms: A patient-unspecific hemodynamics change perspective,” J. Neurointerventional Surg. 8, 1288–1293 (2016). 10.1136/neurintsurg-2015-012154 26880724

[16] N. Gorring , L. Kark , A. Simmons , and T. Barber , “ Determining possible thrombus sites in an extracorporeal device, using computational fluid dynamics-derived relative residence time,” Comput. Methods Biomech. Biomed. Eng. 18, 628–634 (2015). 10.1080/10255842.2013.826655 24460127

[17] D. R. de Sousa , C. Vallecilla , K. Chodzynski , R. C. Jerez , O. Malaspinas , O. F. Eker , R. Ouared , L. Vanhamme , A. Legrand , B. Chopard *et al.*, “ Determination of a shear rate threshold for thrombus formation in intracranial aneurysms,” J. Neurointerventional Surg. 8, 853–858 (2016). 10.1136/neurintsurg-2015-011737 26215274

[18] J. J. Hathcock , “ Flow effects on coagulation and thrombosis,” Arterioscler., Thromb., Vasc. Biol. 26, 1729–1737 (2006). 10.1161/01.ATV.0000229658.76797.30 16741150

[19] M. H. Vlak , G. J. Rinkel , P. Greebe , and A. Algra , “ Independent risk factors for intracranial aneurysms and their joint effect: A case-control study,” Stroke 44, 984–987 (2013). 10.1161/STROKEAHA.111.000329 23422088

[20] S. Kotikoski , J. Huttunen , T. J. Huttunen , K. Helin , J. Frösen , T. Koivisto , M. I. Kurki , M. von und zu Fraunberg , I. Kunnamo , J. E. Jääskeläinen *et al.*, “ Secondary hypertension in patients with saccular intracranial aneurysm disease: A population based study,” PLoS One 13, e0206432 (2018). 10.1371/journal.pone.0206432 30379949 PMC6209332

[21] W. Brinjikji , G. Lanzino , H. Cloft , A. Siddiqui , E. Boccardi , S. Cekirge , D. Fiorella , R. Hanel , P. Jabbour , E. Levy *et al.*, “ Risk factors for ischemic complications following pipeline embolization device treatment of intracranial aneurysms: Results from the intreped study,” Am. J. Neuroradiol. 37, 1673–1678 (2016). 10.3174/ajnr.A4807 27102308 PMC7984677

[22] T. Lassila , A. Sarrami-Foroushani , S. Hejazi , and A. F. Frangi , “ Population-specific modelling of between/within-subject flow variability in the carotid arteries of the elderly,” Int. J. Numer. Methods Biomed. Eng. 36, e3271 (2020). 10.1002/cnm.3271 31691518

[23] G. Mader , M. Olufsen , and A. Mahdi , “ Modeling cerebral blood flow velocity during orthostatic stress,” Ann. Biomed. Eng. 43, 1748–1758 (2015). 10.1007/s10439-014-1220-4 25549771

[24] R. W. Baumgartner , H. P. Mattle , K. Kothbauer , and G. Schroth , “ Transcranial color-coded duplex sonography in cerebral aneurysms,” Stroke 25, 2429–2434 (1994). 10.1161/01.STR.25.12.2429 7974585

[25] V. Nurminen , M. Lehecka , A. Chakrabarty , R. Kivisaari , H. Lehto , M. Niemelä , and J. Hernesniemi , “ Anatomy and morphology of giant aneurysms-angiographic study of 125 consecutive cases,” Acta Neurochir. 156, 1–10 (2014). 10.1007/s00701-013-1933-4 24249668

[26] I. Saatci , H. S. Cekirge , E. F. Ciceri , M. E. Mawad , A. G. Pamuk , and A. Besim , “ CT and MR imaging findings and their implications in the follow-up of patients with intracranial aneurysms treated with endosaccular occlusion with onyx,” Am. J. Neuroradiol. 24, 567–578 (2003). 12695183 PMC8148677

[27] A. Scerrati , G. Sabatino , G. M. Della Pepa , A. Albanese , E. Marchese , A. Puca , A. Olivi , and C. L. Sturiale , “ Treatment and outcome of thrombosed aneurysms of the middle cerebral artery: Institutional experience and a systematic review,” Neurosurg. Rev. 42, 649–661 (2019). 10.1007/s10143-018-0984-7 29790066

[28] L. Pierot , T. Liebig , V. Sychra , K. Kadziolka , F. Dorn , C. Strasilla , C. Kabbasch , and J. Klisch , “ Intrasaccular flow-disruption treatment of intracranial aneurysms: Preliminary results of a multicenter clinical study,” Am. J. Neuroradiol. 33, 1232–1238 (2012). 10.3174/ajnr.A3191 22678844 PMC7965497

[29] A. Sarrami-Foroushani , T. Lassila , A. Gooya , A. J. Geers , and A. F. Frangi , “ Uncertainty quantification of wall shear stress in intracranial aneurysms using a data-driven statistical model of systemic blood flow variability,” J. Biomech. 49, 3815–3823 (2016). 10.1016/j.jbiomech.2016.10.005 28573970

[30] M. Korja , R. Kivisaari , B. R. Jahromi , and H. Lehto , “ Size and location of ruptured intracranial aneurysms: Consecutive series of 1993 hospital-admitted patients,” J. Neurosurg. 127, 748–753 (2016). 10.3171/2016.9.JNS161085 27911237

[31] M. T. Lawton , A. Quiñones-Hinojosa , E. F. Chang , and T. Yu , “ Thrombotic intracranial aneurysms: Classification scheme and management strategies in 68 patients,” Neurosurgery 56, 441–454 (2005). 10.1227/01.NEU.0000153927.70897.A2 15730569

[32] E. Leemans , B. Cornelissen , G. Rosalini , D. Verbaan , J. Schneiders , R. van den Berg , W. Vandertop , E. van Bavel , C. Slump , C. Majoie *et al.*, “ Impact of intracranial aneurysm morphology and rupture status on the particle residence time,” J. Neuroimaging 29, 487–492 (2019). 10.1111/jon.12618 31002750 PMC6618041

[33] L. M. Marsh , M. C. Barbour , V. K. Chivukula , F. Chassagne , C. M. Kelly , S. H. Levy , L. J. Kim , M. R. Levitt , and A. Aliseda , “ Platelet dynamics and hemodynamics of cerebral aneurysms treated with flow-diverting stents,” Ann. Biomed. Eng. 48, 490–501 (2020). 10.1007/s10439-019-02368-0 31549329 PMC7837281

[34] M. M. S. Reza and A. Arzani , “ A critical comparison of different residence time measures in aneurysms,” J. Biomech. 88, 122–129 (2019). 10.1016/j.jbiomech.2019.03.028 30954250

[35] E. Nyilas , W. Morton , D. Lederman , T. Chiu , and R. Cumming , “ Interdependence of hemodynamic and surface parameters in thrombosis,” ASAIO J. 21, 55–70 (1975). 1146031

[36] B. G. Thompson , R. D. Brown, Jr. , S. Amin-Hanjani , J. P. Broderick , K. M. Cockroft , E. S. Connolly, Jr. , G. R. Duckwiler , C. C. Harris , V. J. Howard , S. C. Johnston *et al.*, “ Guidelines for the management of patients with unruptured intracranial aneurysms: A guideline for healthcare professionals from the American Heart Association/American Stroke association,” Stroke 46, 2368–2400 (2015). 10.1161/STR.0000000000000070 26089327

[37] J. Iavindrasana , L. L. Iacono , H. Müller , I. Periz , P. Summers , J. Wright , C. M. Friedrich , H. Dach , T. Gattermayer , G. Engelbrecht *et al.*, “ The@ neurist project,” Stud. Health Technol. Inf. 138, 161–164 (2008). 18560117

[38] M. Villa-Uriol , G. Berti , D. Hose , A. Marzo , A. Chiarini , J. Penrose , J. Pozo , J. Schmidt , P. Singh , R. Lycett *et al.*, “ @ neurist complex information processing toolchain for the integrated management of cerebral aneurysms,” Interface Focus 1, 308–319 (2011). 10.1098/rsfs.2010.0033 22670202 PMC3262441

[39] F. Lin , Y. Xia , S. Song , N. Ravikumar , and A. F. Frangi , “ High-throughput 3dra segmentation of brain vasculature and aneurysms using deep learning,” Comput. Methods Programs Biomed. 230, 107355 (2023). 10.1016/j.cmpb.2023.107355 36709557

[40] L. Shamseer , D. Moher , M. Clarke , D. Ghersi , and A. Liberati , “ Research methods and reporting,” BMJ 349, g7647 (2015). 10.1136/bmj.g7647 25555855

[41] J. Xiang , A. Siddiqui , and H. Meng , “ The effect of inlet waveforms on computational hemodynamics of patient-specific intracranial aneurysms,” J. Biomech. 47, 3882–3890 (2014). 10.1016/j.jbiomech.2014.09.034 25446264 PMC4261154

[42] A. Geers , I. Larrabide , H. Morales , and A. Frangi , “ Approximating hemodynamics of cerebral aneurysms with steady flow simulations,” J. Biomech. 47, 178–185 (2014). 10.1016/j.jbiomech.2013.09.033 24262847

[43] T. Bowker , P. Watton , P. Summers , J. Byrne , and Y. Ventikos , “ Rest versus exercise hemodynamics for middle cerebral artery aneurysms: A computational study,” Am. J. Neuroradiol. 31, 317–323 (2010). 10.3174/ajnr.A1797 19959776 PMC7964156

[44] M. D. Ford , N. Alperin , S. H. Lee , D. W. Holdsworth , and D. A. Steinman , “ Characterization of volumetric flow rate waveforms in the normal internal carotid and vertebral arteries,” Physiol. Meas. 26, 477 (2005). 10.1088/0967-3334/26/4/013 15886442

[45] C. W. Yancy , M. Jessup , B. Bozkurt , J. Butler , D. E. Casey, Jr. , M. M. Colvin , M. H. Drazner , G. S. Filippatos , G. C. Fonarow , M. M. Givertz *et al.*, “ 2017 ACC/AHA/HFSA focused update of the 2013 ACCF/AHA guideline for the management of heart failure: A report of the American College of Cardiology/American Heart Association task force on clinical practice guidelines and the heart failure society of America,” J. Am. College Cardiol. 70, 776–803 (2017). 10.1016/j.jacc.2017.04.025 28461007

[46] S. Ogoh , P. J. Fadel , R. Zhang , C. Selmer , Ø. Jans , N. H. Secher , and P. B. Raven , “ Middle cerebral artery flow velocity and pulse pressure during dynamic exercise in humans,” Am. J. Physiol.-Heart Circ. Physiol. 288, H1526–H1531 (2005). 10.1152/ajpheart.00979.2004 15591094

